# The Firmicutes/Bacteroidetes Ratio as a Risk Factor of Breast Cancer

**DOI:** 10.3390/jcm12062216

**Published:** 2023-03-13

**Authors:** Jeongshin An, Hyungju Kwon, Young Ju Kim

**Affiliations:** 1 Institute of Convergence Medicine Research, Ewha Womans University Mokdong Hospital, College of Medicine, Ewha Womans University, 1071 Anyangcheon-ro, Yangcheon-gu, Seoul 07985, Republic of Korea; 2 Department of Surgery, Ewha Womans University Mokdong Hospital, College of Medicine, Ewha Womans University, 1071 Anyangcheon-ro, Yangcheon-gu, Seoul 07985, Republic of Korea; 3 Department of Obstetrics and Gynecology, Ewha Medical Institute and College of Medicine, Ewha Womans University, Seoul 07804, Republic of Korea

**Keywords:** Firmicutes/Bacteroides ratio, breast cancer, microbiome

## Abstract

The gut microbiome can reflect the health condition of the entire body. Firmicutes and Bacteroidetes, the major phyla of the colon, can influence diseases related to obesity which are also risk factors for breast cancer. Therefore, the Firmicutes/Bacteroidetes (F/B) ratio was analyzed in patients with breast cancer. Bacterial extracellular vesicles were extracted from the serum of patients with breast cancer and healthy controls. Phyla Firmicutes and Bacteroidetes were analyzed using microbiome sequencing. Prognostic factors for breast cancer and serological test results were analyzed for correlations with the F/B ratio. The F/B ratio was three times lower in patients with breast cancer than in healthy controls. In addition, the risk factor for breast cancer, such as fasting serum glucose, was found to be related to the F/B ratio. The F/B ratio can be used as a risk factor of breast cancer and as a clue to explain underlying mechanisms affecting the development of breast cancer.

## 1. Introduction

Breast cancer is the most common female cancer worldwide, and also accounts for the largest proportion of female mortality [1]. Although other cancer types have gradually decreased in frequency with improved hygiene practices or vaccine development, such as gastric cancer and cervical cancer, the incidence of breast cancer is increasing worldwide [2,3]. Genetic factors account for 5–10% of breast cancers, and other contributors include dysregulated female hormones, poor eating habits, and lifestyle [4,5]. Postmenopausal obesity, hyperglycemia, diabetes, hypertension, and metabolic diseases that cause obesity are all known risk factors for breast cancer [6]. Although genetic issues cannot be controlled, controlling obesity and other variable factors may reduce the risk of breast cancer. Obesity is closely related to changes in the gut microbiota [7], and disruption of the gut microbiota can lead to weight gain. In particular, obesity affects the ratio of Firmicutes and Bacteroidetes. Given these associations, differences in F/B ratios may help to identify patients at risk of breast cancer.

The Firmicutes/Bacteroidetes (F/B) ratio is known to have an important effect on maintaining gut homeostasis [8]. In healthy people, the F/B ratio increases significantly with old age [9], and is significantly higher in women than in men [10]. When the F/B ratio is imbalanced, various diseases such as inflammation, autoimmune disease, and cancer can occur. For example, obesity shows a high F/B ratio and inflammatory bowel disease (IBD) shows a low F/B ratio [8]. The F/B ratio was higher in the benign prostatic hyperplasia (BPH) group than in the group without BPH [11]. The F/B ratio is lower in non-alcoholic fatty liver disease (NAFLD)/nonalcoholic steatohepatitis (NASH) patients than in healthy controls [12]. In addition, a higher F/B ratio and lower level of the genus *Bacteroides* were correlated with an increased left atrial diameter [13]. In pregnant women, the F/B ratio was higher in the gestational diabetes mellitus (GDM) group compared to non-GDM group [14]. In summary, the F/B ratio is closely related to health, and this study intends to investigate its correlations with breast cancer.

The breasts are distant from the colon, and it is assumed that the colon cannot directly affect breast disease. However, 99% of the microbial mass present in the human body exists in the gastrointestinal tract, and symbiotic microbiota in the colon affects the entire body via various bacterial metabolites [15,16]. Half of all metabolites in plasma are of bacterial origin [16]. Bacterial extracellular vesicles (EVs) circulate through all body fluids, including blood, lymphatic fluid, amniotic fluid, and breast milk [17]. Bacterial metabolites circulating throughout the body can be attributed to EVs produced by microorganisms, and EVs may influence host pathology through direct or indirect interactions with host cells [18]. We conducted a study to examine how bacterial EVs reflect the activity of symbiotic microbiota using plasma from healthy controls and breast cancer patients, and aimed to identify their relationships to breast cancer and the F/B ratio.

## 2. Materials and Methods

### 2.1. Patients Recruitment and Ethics Statement

This study was conducted with female patients with breast cancer or healthy controls from the Ewha Womans University Mokdong Hospital and Haeundae Paik Hospital. The healthy controls consisted of individuals without a diagnosed disease including diabetes mellitus or alcoholics, and the breast cancer group included patients with stage 0-III breast cancer. This study was conducted as a sub-topic of another study, in the same way as a previous study [19]. In the breast cancer patient group, blood was collected before breast cancer treatment initiation. The exclusion criteria were male sex or use of antibiotics one month before blood sampling. The study was approved by the Institutional Review Board (approval numbers: EUMC 2014-10-005-019 [Ewha Womans University Mokdong Hospital] and 1297992-2015-064 [Inje University Haeundae Hospital]).

### 2.2. EV Isolation and DNA Extraction from Blood

Subjects who consented to participate in the study were enrolled, and blood samples were collected into vacutainer serum separator tubes. Extracellular vesicles (EVs) were collected from the blood as follows. The serum was centrifuged at 1500× *g* at 4 °C for 15 min and diluted in 1× phosphate-buffered saline (PBS, pH 7.4, ML008-01; Welgene, Gyeongsan, Republic of Korea). Thereafter, centrifugation was done at 10,000× *g* at 4 °C for 1 min, followed by filtration using a 0.22 μm filter. Ultraculation was used to obtain EVs. The obtained supernatant was subjected to ultracentrifugation at 150,000× *g* for 3 h at 4 °C in a 45 Ti rotor (Beckman Instruments, Brea, CA, USA). The obtained EV pellet was diluted in PBS and stored at −80 °C. A DNA isolation kit (MoBio PowerSoil DNA Isolation Kit; Qiagen, Hilden, Germany) were used for DNA extraction from EVs [20]. The extracted DNA was quantified using the QIAxpert system (QIAGEN, Hilden, Germany).

### 2.3. Next-Generation Sequencing and Microbiome Analysis

Next-generation sequencing (NGS) was performed using bacteria-specific 16S ribosomal DNA (rDNA). The V3–V4 region of 16S ribosomal DNA was used along with primers, as reported in previous studies [19,20]. The primer sequences were as follows: 16S_V3_F (5′-TCGTCGGCAGCGTCAGATGTGTATAAGAGACAGCCTACGGGNGGCWGCAG-3′) and 16S_V4_R (5′-GTCTCGTGGGCTCGGAGATGTATAAGAGACAGGAC-TACHVGGGTATCTAATCC-3′). A MiSeq system and applications (Illumina, San Diego, CA, USA) were used for 16s metagenomics. NGS library preparation were done according to the Methods guide, and each amplicon was sequenced according to the instructions. A profiling program (MDx-Pro ver.1, MD Healthcare, Seoul, Republic of Korea) was used for taxonomic assignment. The read length was set to 300 bp and the mean quality score was set to >20. High-quality reads were obtained. The CD-HIT sequence clustering algorithm was applied to operational taxonomic units, and UCLUST and QIIME were used for taxonomic assignment. Results were analyzed from the phylum to genus levels. Firmicutes and Bacteroidetes, identified at the phylum level, were used to determine the F/B ratio. In particular, the microbiome, which is occupied largely by the two phyla, was characterized and visualized with circle and bar graphs.

### 2.4. Analysis of Firmicutes/Bateroidetes Ratio in Patients with Breast Cancer

The F/B ratio was obtained by dividing the number of Firmicutes by the number of Bacteroidetes. The relationships of the F/B ratio with body mass index (BMI) and the dietary habits of patients with breast cancer, as well as cancer subtype, stage, and family history, were analyzed. BMI standards differ between countries; therefore, WHO criteria were applied. Fasting serum glucose and hemoglobin levels were analyzed in relation to the F/B ratio. The relationship between the amount of blood oxygen and F/B ratio was analyzed as a possible indicator of the ratio of aerobic and anaerobic bacteria. The ratio of symbiotic bacteria to glucose levels was also analyzed because hyperglycemia is a risk factor for breast cancer. Data were analyzed using the Statistical Package for Social Sciences (SPSS) version 23 (IBM Corp., Armonk, NY, USA) and Prism program version 9.5.1 (733) (GraphPad Software, San Diego, CA, USA). The Student *t*-test was performed for comparison of each group and the receiver operating characteristic (ROC) curve and cutoff value were used to estimate the diagnostic performance of the F/B ratio. The optimal cutoff point was selected based on the Youden’s index. Statistical significance was considered at a two-tailed *p*-value.

## 3. Results

### 3.1. Patients Characteristics

A total of 190 healthy controls and 95 patients with breast cancer were recruited, and age adjustment was performed. This study used data from healthy controls with twice the number of patients with breast cancer. In other words, one patient with breast cancer and two healthy controls of similar age were assigned. The average age of the healthy control group was 51.4 years, and that of the breast cancer patients was 51 years (Table 1). Regarding breast cancer subtypes, luminal cancers accounted for 68% patients, and stage I was the most common. When all cancers were included, the family history of breast and ovarian cancer was 15.7% in third-degree relatives, and an additional 22.1% of patients had a family history of a non-breast/ovarian cancer in third-degree relatives. Serum hemoglobin and glucose levels were obtained within one week before blood collection for microbiome analysis. The mean glucose level was 100 mg/dL and the mean hemoglobin level was 13 g/dL. The mean BMI of all patients with breast cancer was 23.2. Questions about eating habits were conducted using a questionnaire at the time of the hospital visit. There were 18 patients that were vegetarians and so did not eat meat and 5 patients who ate a meat-based diet at every meal (Table 2).

### 3.2. Characteristics of Phyla Firmicutes and Bacteroidetes

**Figure 1:**
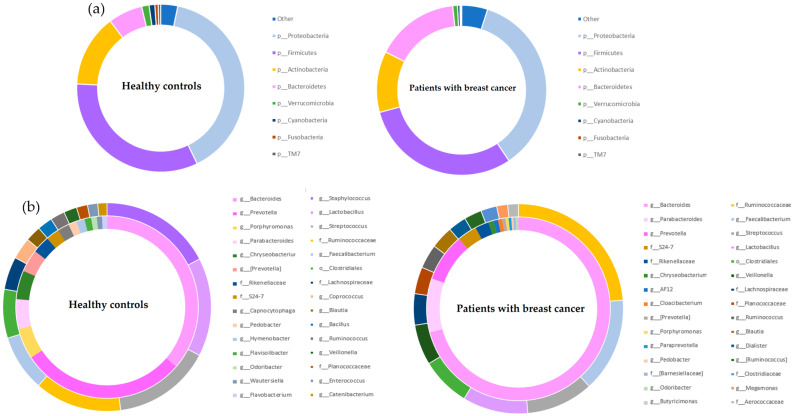
Phyla proportions of total microbiome in healthy controls and breast cancer patients. (**a**) Graph listing phyla-specific abundances in healthy controls and breast cancer. (**b**) Genera-specific abundances in healthy controls and breast cancer. Inner circles show genera belonging to Bacteroidetes, and outer circles show genera belonging to Firmicutes.

This study compared microbiomes between healthy controls and breast cancer patients to investigate possible relationships between Firmicutes and Bacteroidetes prevalence and breast cancer. Age adjustment was performed between groups because age can affect the F/B ratio [8,9]. Alpha and beta diversity assessments were performed between healthy controls and patients with breast cancer (Figure S1). The sum of the top four most prevalent phyla accounted for >95% of the total microbiome in both groups. At the phylum level, Proteobacteria was the most common group, accounting for 39.6% of the normal group microbiota and 39.2% of the breast cancer group microbiota. The next most common group was Firmicutes, accounting for 32.4% of total microbiota in the healthy control group and 30.0% of total microbiota in the breast cancer group. Bacteroidetes were the fourth most common group, accounting for 6.8% and 15.7% of the total microbiota in the health control and breast cancer groups, respectively (Figure 1a).

**Figure 2:**
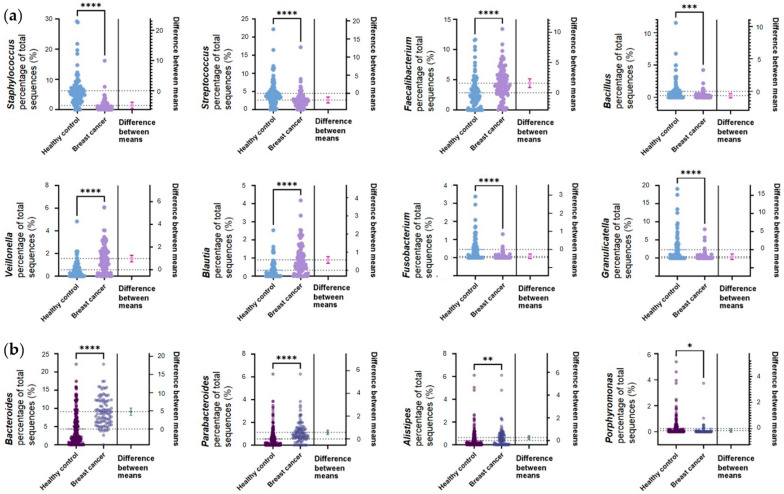
Microbiome differences of the Firmicutes and Bacteroidetes genus-level abundances between healthy controls and patients with breast cancer. (**a**) Statistically significant differentially abundant bacteria from genera of Firmicutes. (**b**) Statistically significant differentially abundant bacteria from genera belonging to Bacteroidetes. * *p* ≤ 0.05, ** *p* ≤ 0.01, *** *p* ≤ 0.001, **** *p* ≤ 0.0001.

The circle graph above shows the abundances of the genera of bacteria belonging to the phyla Firmicutes and Bacteroidetes. The inner circle shows the genera belonging to Bacteroidetes, and the outer circle shows the genera belonging to Firmicutes (Figure 1b). The most common genus of Bacteroidetes was *Bacteroides* in both the healthy control and breast cancer groups, followed by *Prevotella* and *Porphylomonas* in the healthy control group and *Parabacteroides* and *Prevotella* in the breast cancer group. Firmicutes was dominated by *Staphylococcus*, *Lactobacillus*, and *Streptococcus* (in order of abundance) in the healthy control group and *Ruminococcaceae* (f), *Faecalibacterium*, and *Streptococcus* (in order of abundance) in the breast cancer group. Statistically significant differences between groups were analyzed with Student’s *t*-test (Figure 2). The upper two rows in Figure 2a are bacteria belonging to the genera Firmicutes. The third row lists the genera belonging to Bacteroidetes. *Staphylococcus*, *Streptococcus*, *Fusobacterium*, and *Granulicatella*, belonging to Firmicutes, were significantly enriched in healthy controls (*p* ≤ 0.0001). *Faecalibacterium*, *Veillonella*, and *Blautia*, belonging to Firmicutes, were significantly enriched in breast cancer (*p* ≤ 0.0001). The predominant bacteria in breast cancer belonging to Bacteroidetes were *Bacteroides* (*p* ≤ 0.0001), *Parabacteroides* (*p* ≤ 0.0001), and *Alisipes* (*p* ≤ 0.01). *Porphyromonas* was more abundant in healthy controls (*p* ≤ 0.05).

### 3.3. Analysis of F/B Ratio in Patients with Breast Cancer

**Figure 3:**
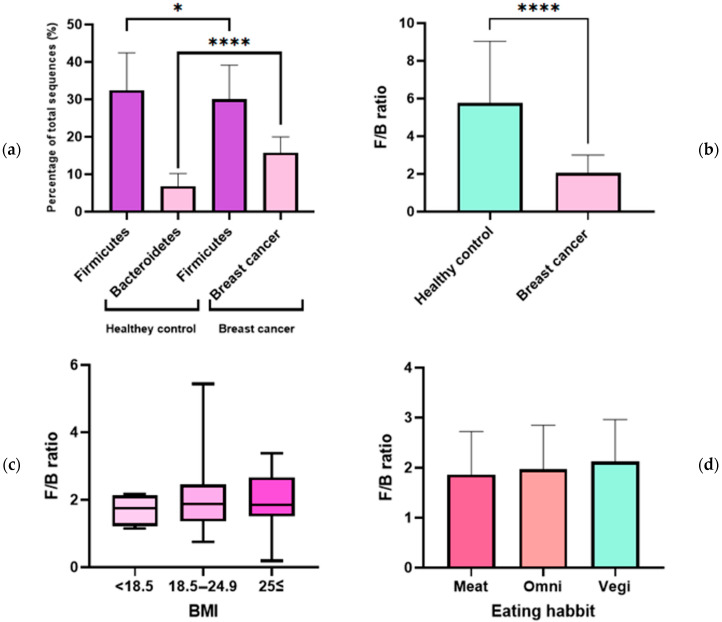
Firmicutes/Bacteroidetes (F/B) ratio in healthy controls and patients with breast cancer. (**a**) Proportion of Firmicutes and Bacteroidetes out of total microbiota in healthy controls and patients with breast cancer. (**b**) F/B ratio in healthy controls and patients with breast cancer. (**c**) The relationship between F/B ratio and body mass index (BMI). BMI in this study was classified according to WHO guidelines (<18.5: Underweight, 18.5–24.9: Normal, 25≤: Overweight or obesity). (**d**) The relationship between F/B ratio and eating habits (Meat: meat-based diet at every meal, Omni: omnivorous, Vegi: vegetarian) * *p* ≤ 0.05, **** *p* ≤ 0.0001.

The ratios of the phyla Firmicutes and Bacteroidetes in healthy controls and patients with breast cancer were analyzed. Firmicutes abundance was significantly different between healthy controls and patients with breast cancer, and was more abundant in healthy controls (*p* ≤ 0.05) (Figure 3a). Bacteroidetes was significantly more abundant in patients with breast cancer (*p* ≤ 0.001) (Figure 3a). The F/B ratio was three times higher in the healthy control group than that of the breast cancer group, and the difference was statistically significant (Figure 3b). The average F/B ratio (2.0) of the healthy control group was considered normal, and those (5.7) of the breast cancer group was considered abnormal. When body mass index (BMI) and F/B ratio were studied in patients with breast cancer, there was no statistically significant correlation; however, the F/B ratio showed a tendency to increase with BMI (Figure 3c). The study results showed a similar pattern to that of a previous study, which found that higher BMIs correlated with higher F/B ratios [21]. Vegetarians tended to have higher F/B ratios than omnivores, and patients who consumed a meat-based diet at every meal had the lowest F/B ratios (Figure 3d).

**Figure 4:**
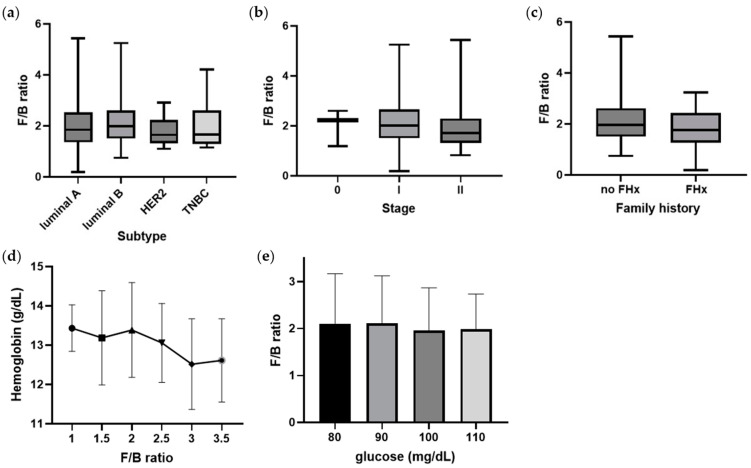
F/B ratio in patients with breast cancer. (**a**) The relationship between F/B ratio and subtypes of breast cancer (luminal A, luminal B, human epidermal growth factor receptor 2 (HER2), or triple negative breast cancer (TNBC)). (**b**) The relationship between F/B ratio and stage of breast cancer. (**c**) The relationship between F/B ratio and family history (FHx). (**d**) The relationship between F/B ratio and hemoglobin level (g/dL). (**e**) The relationship between F/B ratio and fasting serum glucose levels (mg/dL).

The luminal subtype had high F/B ratios, while HER2 and triple negative breast cancer (TNBC) had relatively low F/B ratios (Figure 4a). Although not statistically significant, there was a tendency for HER2 and TNBC with poor prognoses to have a low F/B ratio (mean < 2.0). The F/B ratio also tended to decrease as cancer stage increased from 0 to II (Figure 4b). In particular, the stage II group had an average F/B ratio of less than 2.0. Similarly, the F/B ratio tended to be lower (mean < 2.0) in the group with a family history of cancer (Figure 4c). Hemoglobin level, which is related to the level of oxygen in the blood, decreases as the F/B ratio increases. The fasting serum glucose level also decreases while the F/B ratio increases. When the blood glucose level was higher than 100 mg/dL, the average F/B ratio was lower than 2.0. However, these correlations between hemoglobin and fasting serum glucose level with F/B ratio were not significant (Figure 4d,e).

**Figure 5:**
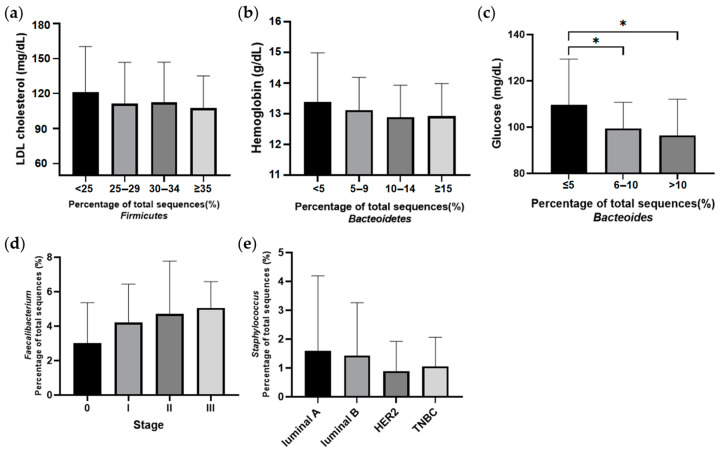
Relationships of specific microbiome member abundance to factors related to breast cancer. (**a**) LDL cholesterol levels (mg/dL) according to abundance of phylum Firmicutes. (**b**) Hemoglobin levels (g/dL) according to abundance of phylum Bacteroidetes. (**c**) Fasting serum glucose levels (mg/dL) according to abundance of genus *Bacteroides*. (**d**) Abundance of genus *Faecalibacterium* according to stage of breast cancer. (**e**) Abundance of genus *Staphylococcus* according to subtypes of breast cancer (luminal A, luminal B, human epidermal growth factor receptor 2 (HER2), or triple negative breast cancer (TNBC)). * *p* < 0.05.

**Figure 6:**
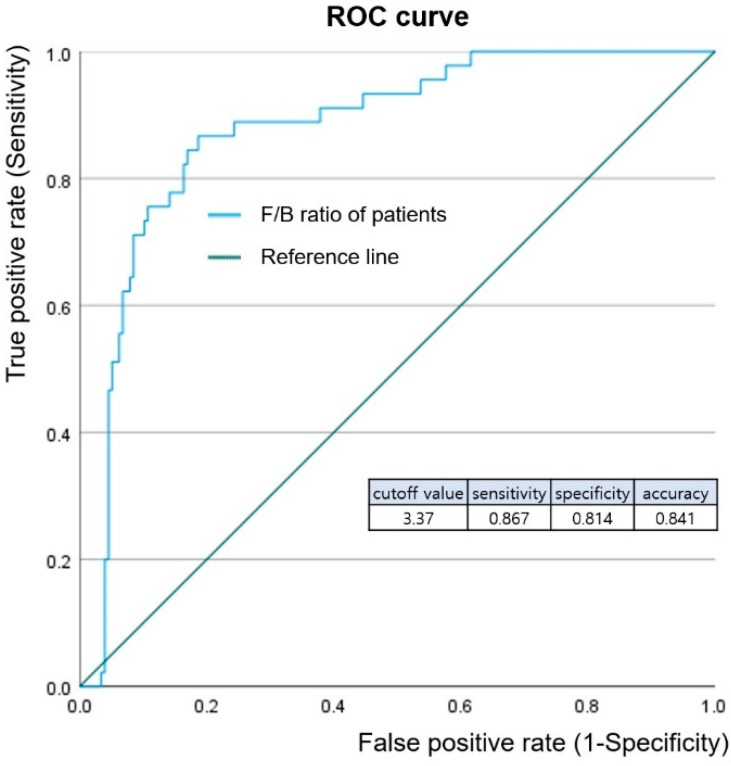
Receiver operating characteristic (ROC) curve analysis of F/B ratio. ROC curve analysis was conducted to diagnosis breast cancer through F/B ratio. The ROC curve of the F/B ratio showed a good distance between patients with breast cancer and healthy controls, with sensitivity of 0.867, specificity of 0.814, and accuracy 0.841.

The genera included in the F/B ratio were found to be different between the breast cancer and healthy control groups (Figure 5). In particular, the most abundant bacteria in the breast cancer group were predominantly anaerobes, regardless of being part of Firmicutes or Bacteroidetes. Firmicutes abundance was relatively low in patients with breast cancer compared to healthy controls. The level of low-density lipoprotein (LDL) cholesterol decreased with increasing amounts of Firmicutes. Higher abundances of Bacteroidetes were related to lower levels of hemoglobin and fasting glucose in the Patients with breast cancer. *Faecalibacterium* abundance was associated with the breast cancer stage, and increased at higher stages compared to lower stages. An ROC curve was drawn to predict the incidence of breast cancer through the F/B ratio, and the optimal cutoff value of the F/B ratio was 3.37 (Figure 6). Breast cancer was identified with a sensitivity of 86.7%, specificity of 81.4%, and accuracy of 84.1% via optimal cutoff value. This cutoff value is higher than the average F/B ratio (2.0) for patients with breast cancer.

## 4. Discussion

The F/B ratio can be used as a biomarker for breast cancer. The F/B ratio is reflective of the balance of intestinal symbiotic microbiota. The F/B ratio has been studied in not only intestinal diseases such as IBD [8], but also in metabolic diseases such as obesity, NAFLD, inflammatory diseases, and cancers [12,22]. Intestinal microbiota can affect the entire body. The balance of these gut microbes can be related to diseases in which bacterial metabolites such as short-chain fatty acids and lipopolysaccharides exert effects on health [23]. Breast cancer is thought to be influenced by symbiotic bacteria from the colon through bacterial EVs via the gut-breast axis. In this study, this pathway was investigated by assessing the composition of the EV microbiome, specifically the F/B ratio. Firmicutes and Bacteroidetes are major members of the gut microbiota. Because microbial metabolites are loaded into EVs and circulate via body fluids, the composition of the F/B ratio of EVs is indirect evidence of this pathway. Each target organ has a specific microbiome ratio that can reflect disease state. For example, diseases of the heart or prostate have specific F/B ratios [11,13]. This was confirmed via sequencing of bacterial EVs in patients with breast cancer. Thus, F/B ratios can be used as an indicator of breast cancer.

The results of this study validate the potential use of F/B ratio in characterizing and/or diagnosing breast cancer. When the F/B ratio pattern was investigated, the F/B ratio was lower in breast cancer subtypes associated with poor prognosis. Human epidermal growth factor receptor 2 (HER2) and triple negative breast cancer (TNBC) have a relatively poor prognosis among breast cancer, and EVs from patients of these subtypes had lower F/B ratios [24,25]. In addition, as stage increases, the prognosis becomes poorer [26]. Although there was no statistical significance, there was a trend of decreased F/B ratio in with increased stage. A family history of breast and ovarian cancer, as well as a family history of other cancers, is implicated in poorer prognosis of breast cancer [27,28]. We found that F/B ratio is lower in patients with a family history than patients without. These data show the potential for F/B ratio to reflect prognosis. The F/B ratio in the serum reflects symbiosis or dysbiosis of symbiotic microbiota, which affects breast cancer via EVs.

The contribution of each specific bacterium may also have important roles in health. The abundance of *Bacteroides* (a member of Bacteroidetes) showed a close correlation with fasting glucose levels in patients with breast cancer. Increased fasting glucose levels are a risk factor for breast cancer [29]. The main energy source of *Bacteroides* is the fermentation of dietary or host-derived glycans [30]. The bacterial compounds from the fermentation of these glycan derivatives are commonly found in the human colon and are potentially toxic [31]. This bacterial product may have an adverse effect on breast cancer prognosis, and higher levels of Bacteroides may be a risk factor for the development of breast cancer. On the other hand, LDL cholesterol levels decreased with increasing abundances of Firmicutes in this study. Many Firmicutes are butyrate producers and play a role in promoting fatty acid absorption [32], which may decrease LDL levels. High LDL cholesterol, which is indicative of dyslipidemia, is a risk factor for breast cancer [33]. A relatively low abundance of Firmicutes is likely to have an adverse effect on breast cancer prognosis. *Faecalibacterium* belongs to the phylum Firmicutes and is abundant in patients with breast cancer (Figure 5). This bacterium is associated with high stage breast cancer. *Faecalibacterium* is a short-chain fatty acids (SCFAs) producing bacteria [34]. SCFAs are important regulators of the microbiome and immune function and are also potent epigenetic modifiers, exhibiting functions such as histone deacetylase (HDAC) inhibitory activity [35]. HDAC inhibitors are potent inducers of growth arrest, differentiation, and death in transformed cells [36]. *Faecalibacterium*, which is more abundant in high stage breast cancer, might be related to the rapid growth of cancer cells and cell death. *Staphylococcus* has been previously studied for its potential involvement in endocrine therapy efficacy for breast cancer [19]. The relative abundance of *Staphylococcus* in the luminal subtypes supports this finding (Figure 5). HER2 and TNBC, which are known to have poor prognosis, tend to have a relative deficiency of *Staphylococcus*. Considering the abundance of *Staphylococcus* in the healthy control group, it can be inferred that a deficiency of *Staphylococcus* may contribute to breast cancer. The effects of the microbiome on breast cancer cannot be generally defined because the proportions of symbiotic microbiota in the colon are important. The F/B ratio can reflect factors such as stage, subtype, family history, and blood test results. Healthy conditions were maintained when the microbiome ratio was balanced. When the balance is disturbed, diseases such as breast cancer can occur [15]. This paper is significant by presenting the cutoff value of the F/B ratio, which is out of balance due to breast cancer. When the F/B ratio is 3.37 or less, it not only means that the balance of microbial symbiosis is broken, but also means that the risk of breast cancer is increased.

So far, the importance of the microbiome of the colon has been emphasized according to previous studies [37,38]. The F/B ratio is mainly applied to diseases occurring spatially close to the colon or metabolic diseases, but this study investigates its application to breast cancer, a distant organ from the colon via blood samples. According to this study, F/B ratio is one of risk factors. Since 50% of metabolites in the human blood are of microbial origin [16], the change of F/B ratio causes the dysbiosis of microbiota, including the unbalance of metabolites in the blood. This dysbiosis is related to eating habits and lifestyle and causes the change of the F/B ratio. Therefore, F/B ratio could be a trigger of one of the disease pathways leading to breast cancer. A risk factor analysis will be continued in a future study. A limitation of this study was the lack of clinical data from a healthy control group. However, the significance of this study lies in defining the optimal cutoff value of the F/B ratio as 3.37. In addition, this study implemented NGS as part of a large project, validated the effects of abnormal F/B ratios, and studied the composition of specific bacteria. If the procedure was simplified using PCR, the F/B ratio can be determined more quickly.

## 5. Conclusions

The relationship between breast cancer and the F/B ratio has the potential to be used in a diagnostic method for breast cancer. In addition, the risk factor for breast cancer is associated with a lower F/B ratio (<2.0), which is related with fasting glucose level and cancer stages. Further studies on more patients could increase the accuracy of the study.

This study can be an important clue regarding diseases described by the F/B ratio. It can also be used as evidence for research predicting the development of breast cancer using the F/B ratio.

## Figures and Tables

**Table 1:** Characteristics of healthy controls and patients with breast cancer.

Characteristics	Healthy Controls	Patients with Breast Cancer
Number of female patients (N)	190	95
Age (years)	51.4 ± 9.7	51.0 ± 10.4

**Table 2:** Characteristics of patients with breast cancer.

Characteristics	Patients with Breast Cancer
Subtype	
Luminal A	30 (31.5%)
Luminal B	38 (40%)
HER2	12 (12.6%)
TNBC	15 (15.7%)
Stage	
0	3 (3.1%)
I	44 (46.3%)
II	35 (36.8%)
III	13 (13.6%)
Family history	
Yes Cancer: Breast, Ovary	15 (15.7%)
Others	21 (22.1%)
no	59 (62.1%)
Hemoglobin	13 ± 1.1 (g/dL)
Glucose	100.1 ± 14.7 (mg/dL)
LDL cholesterol	113.0 ± 34.3 (mg/dL)
BMI	
~19	18 (18.9%)
20~24	50 (52.6%)
25~29	21 (22.1%)
30~	6 (6.3%)
Eating habits	
omnivorous	64 (67.3%)
vegetarian	18 (18.9%)
meat based diet	5 (5.2%)
nonresponse	8 (8.4%)

HER2 (human epidermal growth factor receptor 2), TNBC (triple-negative breast cancer), LDL (low-density lipoprotein), body mass index (BMI).

## Supplementary Materials

The following supporting information can be downloaded at: https://www.mdpi.com/article/10.3390/jcm12062216/s1, Figure S1: Alpha and beta diversity between healthy controls and patients with breast cancer.
