# Supplementary material for: The Firmicutes/Bacteroidetes Ratio as a Risk Factor of Breast Cancer
Source: J Clin Med. 2023 Mar 13;12(6):2216. doi: 10.3390/jcm12062216 (PMC10052522; doi:10.3390/jcm12062216)
Supplement: Supplementary file 1 [file jcm-12-02216-s001.zip › jcm-2090405-supplementary.pdf]

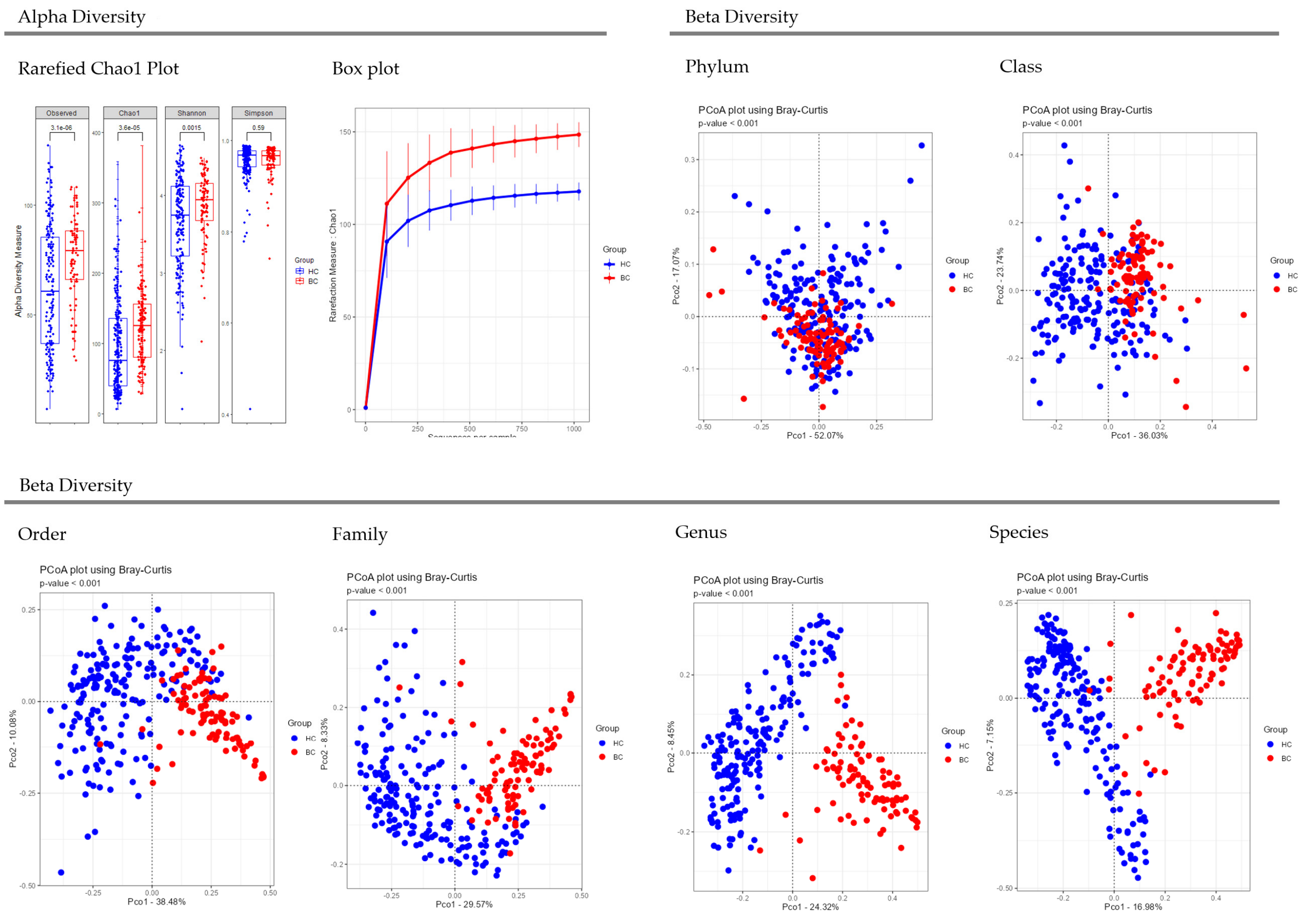

Figure S1. Alpha and beta diversity between healthy controls and patients with breast cancer.
